# *Pseudomonas aeruginosa* PA1006 Is a Persulfide-Modified Protein That Is Critical for Molybdenum Homeostasis

**DOI:** 10.1371/journal.pone.0055593

**Published:** 2013-02-08

**Authors:** Gregory Tombline, Johanna M. Schwingel, John D. Lapek, Alan E. Friedman, Thomas Darrah, Michael Maguire, Nadine E. Van Alst, Melanie J. Filiatrault, Barbara H. Iglewski

**Affiliations:** 1 Department of Microbiology and Immunology, University of Rochester School of Medicine and Dentistry, Rochester, New York, United States of America; 2 Department of Environmental Medicine, University of Rochester School of Medicine and Dentistry, Rochester, New York, United States of America; 3 Division of Earth and Ocean Sciences, Nicholas School of the Environment, Duke University, Durham, North Carolina, United States of America; Centre National de la Recherche Scientifique, Aix-Marseille Université, France

## Abstract

A companion manuscript revealed that deletion of the *Pseudomonas aeruginosa (Pae)* PA1006 gene caused pleiotropic defects in metabolism including a loss of all nitrate reductase activities, biofilm maturation, and virulence. Herein, several complementary approaches indicate that PA1006 protein serves as a persulfide-modified protein that is critical for molybdenum homeostasis in *Pae*. Mutation of a highly conserved Cys22 to Ala or Ser resulted in a loss of PA1006 activity. Yeast-two-hybrid and a green-fluorescent protein fragment complementation assay (GFP-PFCA) in *Pae* itself revealed that PA1006 interacts with *Pae* PA3667/CsdA and PA3814/IscS Cys desulfurase enzymes. Fourier transform ion cyclotron resonance mass spectrometry (FT-ICR-MS) “top-down” analysis of PA1006 purified from *Pae* revealed that conserved Cys22 is post-translationally modified *in vivo* in the form a persulfide. Inductively-coupled-plasma (ICP)-MS analysis of Δ*PA1006* mutant extracts revealed that the mutant cells contain significantly reduced levels of molybdenum compared to wild-type. GFP-PFCA also revealed that PA1006 interacts with several molybdenum cofactor (MoCo) biosynthesis proteins as well as nitrate reductase maturation factor NarJ and component NarH. These data indicate that a loss of PA1006 protein’s persulfide sulfur and a reduced availability of molybdenum contribute to the phenotype of a Δ*PA1006* mutant.

## Introduction

*Pseudomonas aeruginosa* (*Pae*) is an opportunistic human pathogen capable of causing many different kinds of infections including localized acute infections of the eye, chronic localized lung infections prevalent in Cystic Fibrosis (CF) patients, as well as disseminated infections such as occurs after severe burns (thermal injury) [1], [2]. *Pae* is also among the most prevalent infections that occur in intensive care units and its incidence is on the rise [3]. Drug resistance is also increasing among *Pae* strains in the clinic, and options to combat it are limited [4]. Thus, there is a pressing need to develop novel strategies to disable this pathogen that will not lead to resistance. One approach to avoiding drug resistance may be to specifically target virulence-specific pathways [5].

*Pae* virulence mechanisms are multifactorial. Several *Pae* virulence factors such as secreted factors, proteases, exotoxins, cyanide, and phenazines contribute to pathogenesis [6]. While an in-depth understanding of *Pae* metabolism during localized eyes infections or in disseminated infections is lacking, transcriptome and metabolome studies of *Pae* clinical isolates obtained from CF patients have begun to emerge. In these cases, long-term infection appears to involve complex physiological adaptations in *Pae* that distinguish them from cells cultured in the laboratory (*in vitro*) in rich media [7], [8]. In a companion study we demonstrate that loss of the PA1006 protein confers pleiotropic defects in metabolism (particularly in nitrate utilization), biofilm formation, and virulence in model organisms [9]. In this study, we set out to provide a mechanistic basis for PA1006 function that will facilitate future drug design efforts.

**Figure 1:**
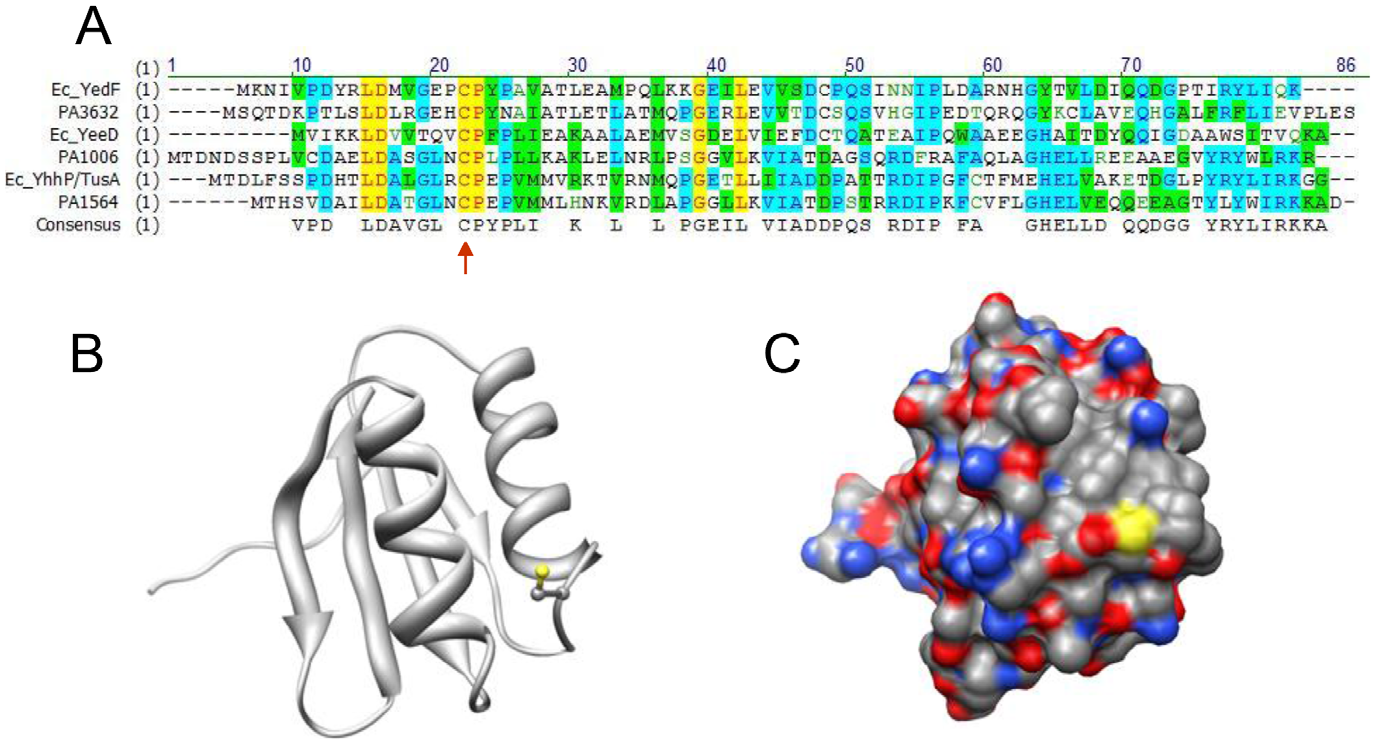
PA1006 is an ortholog of the *E. coli* YhhP/TusA protein that functions in sulfur trafficking. (A) Alignment of *Pae* and *E. coli* orthologs of PA1006. Alignment was performed with VectorNTI software using default parameters (Clustal W). B and C) Homology model of PA1006 shown as ribbon or space-filling (with element coloring scheme) representations. The highly conserved Cys22 is shown in the ribbon view and its sulfur (yellow) is also apparent in the space-filling view. Model was generated by SwissModel webserver [16] and displayed using UCSF Chimera [17], [18].

The *PA1006* gene encodes a conserved hypothetical protein of 85 amino acids; yet, its precise function cannot be easily deciphered using a bioinformatic approach. *E. coli* and *Pae* each possess at least three orthologs of PA1006 protein (Fig. 1). In *E. coli* they are encoded by the *yhhP/tusA*, *yedF*, and *yeeD* genes. In *Pae*, in addition to *PA1006*, *PA1564* and *PA3632* are also present. These proteins do not appear to be functionally redundant in either organism since mutation of either the *yhhP/tusA* gene in *E. coli* [10], [11] or *PA1006* gene in *Pae* [9]elicits a phenotype that is not suppressed by the other orthologs. Moreover, unlike *PA1006*, *PA1564* appears to be essential in *Pae* strain PAO1 since several attempts to delete it failed, and *PA3632* is not required for anaerobic growth with nitrate (data not shown).

PA1006 orthologs appear to be present in many, but not all, bacteria including several pathogens. Only two eukaryotic orthologs were found: in *Oryza sativa* (japonica rice) and *Nematosella vectensis* (sea anemone). The structure of the *E. coli* YhhP/TusA protein was determined by NMR [12] and the Structural Classification of Protein (SCOP) database [13], [14] classifies the YhhP/TusA structure as displaying an IF3-like fold (translation Initiation Factor-3) consisting of two alpha helices that rest upon four beta strands (beta-alpha-beta-alpha-beta2). This fold occurs in several protein superfamilies including the C-terminal domains of IF3 and ProRS, YhbY, SirA, AlbA, RH3, and EPT/RTPC proteins.

A highly conserved cystein (Cys) in PA1006 (residue 22) as well as homology to the *E. coli* YhhP/TusA protein may provide insight into its biological role (Fig. 1A, B). Genetic and biochemical studies tracing the origins of 2-thiouridine modifications of *E. coli* tRNAs earned YhhP/TusA the alternate name of “TusA*”* or, tRNA uridine sulfuration protein A [10]. In 2-thiouridine biogenesis, the highly conserved Cys residue of YhhP/TusA functions as a persulfide sulfur carrier. The persulfide sulfur originates from the Cys desulfurase enzyme IscS. IscS converts Cys to Ala, liberating the sulfur by first forming a persulfide on itself. Next, IscS passes the persulfide to the highly conserved Cys of YhhP/TusA. Subsequently, YhhP/TusA passes the persulfide sulfur to a Cys residue within another set of relay proteins called TusBCD [11]. Eventually, this persulfide sulfur atom is transferred to tRNA forming a 2′-thiouridine group [11]. YhhP/TusA -related biochemical studies indicating a role in sulfur trafficking were also supported by reciprocal pairwise interactions found between IscS and YhhP/TusA in comprehensive protein-protein interaction network mapping experiments performed in *E. coli* [15]. Tagged versions of either YhhP/TusA or IscS proteins were used as “bait” and the reciprocal protein was “pulled-down” and identified by mass spectrometry.

Given the strong conservation of PA1006 Cys22, we hypothesized that PA1006 may also be modified with a persulfide at this position and that this may be critical for its biological role. A secondary hypothesis we pursued is that PA1006 protein may be connected to molybdenum-dependent pathways since, in addition to the loss of nitrate reductase activities which require MoCo, several MoCo biosynthesis genes showed altered expression in the Δ*PA1006* mutant [9].

## Results

### Mutagenesis of the Highly Conserved Cys22 of PA1006

A hallmark of *E. coli* YhhP/TusA or *Pae* PA1006 protein orthologs is a highly conserved Cys residue (Fig. 1A; red arrow). In YhhP/TusA, the equivalent conserved Cys accepts a sulfur atom donated from the Cys desulfurase IscS which is then relayed to become 2′-thiouridine-tRNA [10]. Since this Cys is conserved in PA1006 (Fig. 1A), we mutated Cys22 to alanine (Ala) or serine (Ser). Ala was chosen because it replaces the sulfhydryl with a methyl group, and abolishes the capacity to carry a persulfide. Ser was chosen because its hydroxyl group similarly negates the ability to carry a persulfide; yet, the hydroxyl may preserve hydrogen bonding potential. Next, we determined if the Cys22Ala or Cys22Ser mutant proteins expressed from a plasmid (pucp18) under the control of the *PA1006* gene’s native promoter were able to complement the nitrate utilization/anaerobic growth defect of the Δ*PA1006* chromosomal deletion mutant [31]. As a positive control for functional complementation, the wild-type (WT) *PA1006* gene was included in parallel. Neither the Cys22Ala nor Cys22Ser mutant was capable of restoring anaerobic growth to the Δ*PA1006* mutant whereas the WT gene restored growth under these conditions; however, western blots showed that mutant and WT displayed similar levels of expression (data not shown). These data highlight the importance of the highly conserved Cys22 for PA1006 function and suggest that Cys22 may function as a persulfide carrier in sulfur trafficking pathways. Figure 1B and C show a homology model of the PA1006 protein (generated with Swiss Model [16] and visualized with UCSF Chimera [17], [18]) based upon the known NMR structure of the *E. coli* YhhP/TusA protein [12] with the highly conserved Cys22 colored in yellow.

### PA1006 Interacts with the Cys Desulfurase PA3667/CsdA and its Partner PA3668/CsdE

Prompted by the interaction of *E.coli* YhhP/TusA with IscS and our C22Ala/Ser mutagenesis data, we employed the Matchmaker yeast-two-hybrid system (Clontech) to test if the PA1006 protein interacts with three proteins that show homology to cysteine desulfurases in *Pae*: PA3814/IscS, PA3667/CsdA, and a putative pyridoxal-phosphate dependent enzyme PA2062. In each case, empty vector controls were paired opposite each inserted fusion to test for false positives caused by activation of a single gene versus either the GAL4-DNA binding domain (GAL4-DBD) or the GAL4-transcription activation domain (GAL4-AD). Although we cannot entirely rule out an interaction in any case, PA3667/CsdA interacted with PA1006 as indicated by growth on selective –Trp-Leu-His triple dropout (TDO) media (Table 1) as well as β-galactosidase (β-gal) activity measured by the colony lift assay (data not shown). Similarly, based upon the same criteria (TDO+/β-gal+), we also found an interaction between PA1006 and PA3668/CsdE. In addition, we found reciprocal pairwise interactions between PA3667/CsdA and PA3668/CsdE which were able to grow on TDO media,–Trp-Leu-His-Ade quadruple dropout (QDO) media, as well as display β-galactosidase activities on colony lift assays. Since interactions between CsdA and CsdE (as well as the closely homologous SufS and SufE) were demonstrated and structurally modeled with *E. coli* proteins [19], [20], the interaction between PA3667/CsdA and PA3668/CsdE provides a good validation of the yeast-two-hybrid method. Interaction of PA1006 with PA3814/IscS could not be entirely ruled out because PA3814/IscS displayed a false positive interaction by itself when paired with the empty GAL4-AD vector. A third potential Cys desulfurase, PA2062, did not show interaction with PA1006 in either orientation on any of the dropout media or by β-galactosidase colony lift assays (data not shown).

**Table 1:** Yeast-Two-Hybrid analysis of PA1006 interactions as indicated by growth on TDO (-Leu/−Trp/−His) media.

Bait (Gal4-DBD fusion)	Prey (Gal4-AD fusion)	Interaction?
PA1006	PA1006	no
PA1006	PA3814/IscS	no
PA1006	PA3667/CsdA	no
PA3814/IscS	PA1006	ND*
PA3667/CsdA	PA1006	yes
PA3667/CsdA	PA3668/CsdE	yes
PA3668/CsdE	PA3667/CsdA	yes
PA1006	PA3668/CsdE	yes
PA3668/CsdE	PA1006	no
PA3667/CsdA	PA1006- C22A	yes
PA3667/CsdA	PA1006-C22S	yes
PA2062	PA1006	no
PA1006	PA2062	no

* Could not be determined because Gal4-DBD-PA3814/IscS displayed a false positive signal when paired with the empty Gal4-AD.

### Purification and Analysis of His_6_-PA1006 from *Pae*

**Figure 2:**
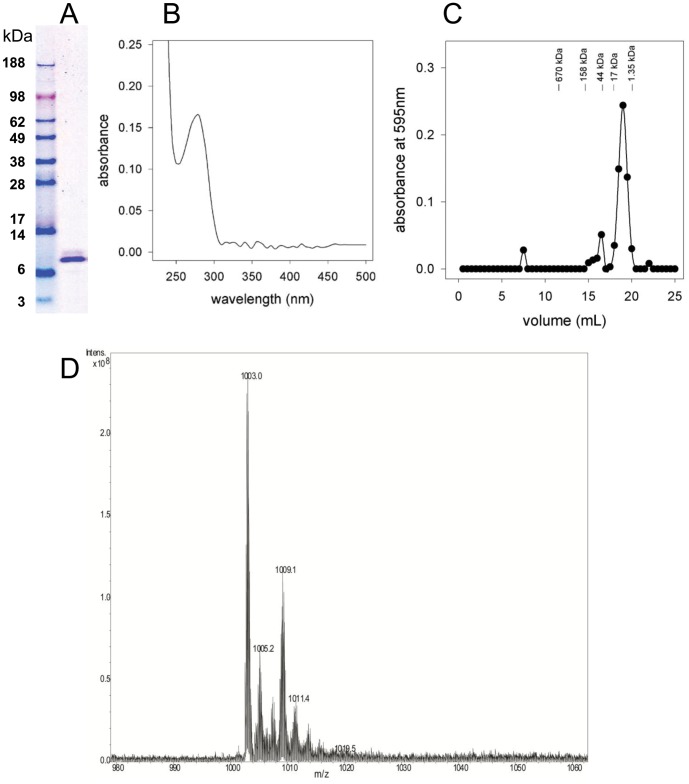
Purification and properties of His_6_-PA1006 from *Pae*. (A) SDS PAGE analysis and (B) absorption spectrum of His_6_-PA1006 purified to near homogeneity from *Pae*. Analytical gel-filtration chromatography (C) shows that pure His_6_-PA1006 is monodisperse and approximates the size of a monomer. (D) FT-ICR-MS analysis of His_6_- PA1006 purified from *Pae* shows at least two major species of m/z 1003 and m/z 1009.

Given the yeast-two-hybrid data, we next tried to demonstrate sulfur transfer between the PA3667/CsdA Cys desulfurase and PA1006 *in vitro*. We were able to purify recombinant His_6_-PA1006 and His_6_-PA3667/CsdA, in high yield to near homogeneity from *E. coli* (Fig. S1). Notably, the absorbance spectrum of His_6_-PA3667/CsdA indicated that it contained a pyridoxal phosphate cofactor (seen at ∼400 nm) as expected (Fig. S1C). Pyridoxal phosphate cofactor (100 µM) was added to buffers during purification and storage in order to preserve the active form of the enzyme. Analytical gel filtration indicated that PA3667/CsdA formed a dimer in solution (Fig. S1D). These data indicated that His_6_-PA3667/CsdA was properly folded and active. While PA3667/CsdA displayed Cys desulfurase activity and was capable of transferring ^35^S (derived from Cys) to itself *in vitro*, we did not observe transfer of sulfur to PA1006 above background levels (Fig. S1D). His_6_-PA3814/IscS was also purified from *E. coli* with similar yield and purity (data not shown). In contrast to PA3667/CsdA, PA3814/IscS failed to show any sulfur transferase activity under any conditions we tried *in vitro*. We surmised that reconstitution of sulfur transfer activity from either PA3667/CsdA or PA3814/IscS to PA1006 *in vitro* may require additional factors that are present *in vivo*. It was because of these initial disappointing attempts to reconstitute sulfur transfer *in vitro* that we decided to purify PA1006 from *Pae* itself and look for a persulfide modification on *in vivo*-derived protein. Specifically, our goal was to use high resolution mass spectrometry to examine Cys22 of PA1006 to see if it carries a persulfide modification. Using appropriate primers in PCR, the *PA1006* gene was engineered to have an extra methionine followed by a glycine and six histidine residues at its amino terminus (encoding a His_6_-PA1006 protein). His_6_-PA1006 was expressed in *Pae* from plasmid pEX1.8 upon induction with isopropyl-1-thio-β-D-galactopyranoside (IPTG). Similar to the pucp18 native *PA1006* gene construct, the His_6_-*PA1006* gene expressed from plasmid pEX1.8 was able to confer anaerobic growth in the Δ*PA1006* mutant strain when grown in the presence of nitrate (data not shown). This result indicates that the His_6_-PA1006 protein is fully functional *in vivo*. Next, His_6_-PA1006 was purified by Ni^2+^-NTA agarose, and remaining contaminants were removed by subsequent passage through a MonoQ (GE Life Sciences) anion exchange column. His_6_-PA1006 protein eluted in the flow-through fractions of the MonoQ column whereas residual contaminants were retained (the same method was also used to purify PA1006 from *E. coli* with similar results). The resulting His_6_-PA1006 obtained from *Pae* was relatively pure as determined by SDS PAGE followed by Coomassie staining and its size corresponds well with the expected ∼10 kDa mass (Fig. 2A). In contrast to His_6_-PA3667/CsdA, the absorption spectrum of purified His_6_-PA1006 shows baseline absorption levels in the 300–500 nm region of the spectrum. This indicates that, unlike PA3667/CsdA, a pyridoxal phosphate cofactor is not tightly associated with PA1006 (Fig. 2B). Resolution by analytical gel-filtration (Superose 6 column, GE Life Sciences) showed that His_6_-PA1006 protein eluted as a monodisperse peak that approximates the size of a monomer; however, a dimer cannot be ruled out due to the resolution of the column (Fig. 2C). The identity of purified His_6_-PA1006 protein was also confirmed by trypsin digestion followed by “bottom-up” mass spectrometry methods (see Methods; data not shown).

### Analysis of His_6_-PA1006 Protein by Fourier Transform Ion Cyclotron Resonance Mass Spectrometry (FT-ICR-MS)

His_6_-PA1006 protein purified from *Pae* was analyzed “top-down” by FT-ICR-MS (Fig. 2D). His_6_-PA1006 protein purified from *Pae* contained two notable (+10) charged species with m/z values of 1003 and 1009. The m/z 1009 species was of particular interest to us because initial experiments (using 60% methanol; 3% acetic acid as the solvent) revealed that this species is acid labile since it dissipated rapidly during the timescale of the analysis. When 60% methanol; 0.1% formic acid was used as the solvent we found the m/z 1009 species to be relatively stable during the timescale of the analysis. In addition, similar m/z 1003 and m/z 1009 species were present when the *Pae* cells were grown in different medias such as Luria broth (LB) or nutrient/yeast extract broth in the absence or presence of 100 mM nitrate (NY or NY+NO_3_; data not shown).

**Figure 3:**
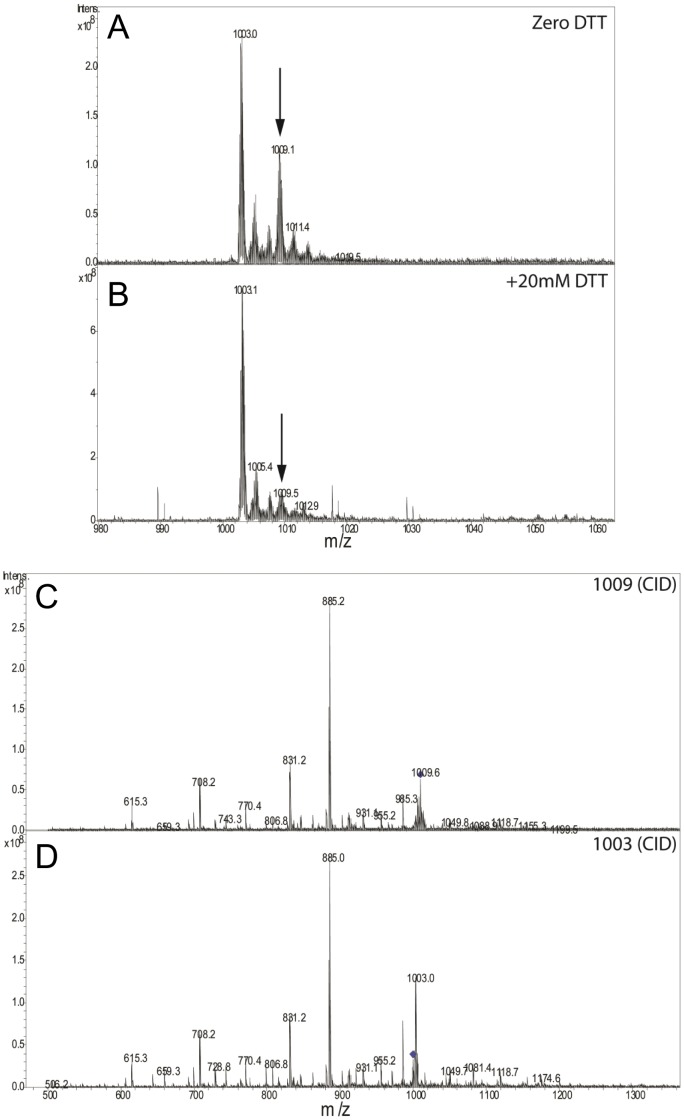
His_6_-PA1006 purified from *Pae* contains a DTT-labile species. FT-ICR-MS analysis of His_6_-PA1006 purified from *Pae* before (A) and after (B) treatment with 20 mM DTT for 20 min. Species m/z 1009 (C) and m/z 1003 (D) were isolated and fragmented by CID showed an identical fragmentation pattern indicated that they are derived from the same initial peptide.

**Figure 4:**
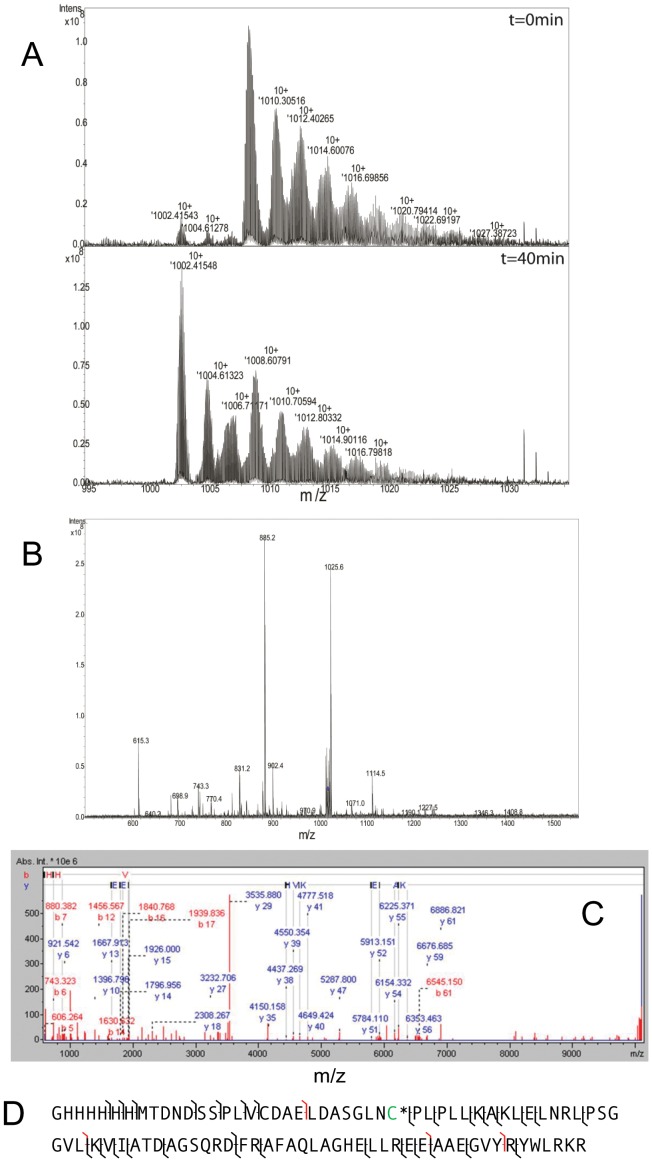
“Top-down” analysis of His_6_-PA1006 by FT-ICR-MS. A) Upper and lower panels show FT-ICR-MS spectra of a recent preparation of His_6_-PA1006 immediately after dilution with 60% methanol; 0.1% formic acid solvent (upper) and after 40 min (lower) of being in the solvent respectively. Data were collected at >100,000 resolution in narrow band mode. B) The m/z 1009 species was isolated and fragmented by CID. The resulting fragments were deconvoluted and masses assigned by Data Analysis in (C). Fragments (“b” and “y” ions) are depicted in (D). Figure S2 shows the constituent fragmented ions in detail.

In order to further interrogate the nature of the m/z 1003 and m/z 1009 species, we performed several experiments. Given that we already suspected that the m/z 1009 peak may represent a persulfide-modified form, we tested the effect of reduction by 20 mM dithiothreitol (DTT) which would remove a persulfide group. Consistent with a persulfide modification, treatment with DTT resulted in the loss of the m/z 1009 species (Fig. 3A and B). Next, the FT-ICR-MS was used to isolate the m/z 1003 and 1009 species separately in the gas phase and each was fragmented into its constituent peptides using collision-induced dissociation (CID). CID fragmentation spectra of m/z 1003 and 1009 species were identical indicating that they contain the same constituent fragments and also indicates that the m/z 1009 species is a post-translationally modified form of the m/z 1003 species (Fig. 3C and D). Given that the m/z 1009 species is sensitive to acid, reducible with DTT, and carries an additional ∼63 Da mass, two additional sulfurs added to His_6_-PA1006 may account for the increased mass compared to the m/z 1003 species. To further evaluate the nature of the m/z 1009 species, we isolated and fragmented this species using a preparation of His_6_-PA1006 from *Pae* that displayed a relatively high ratio of m/z 1009∶1003 species. At the same time, we also minimized the exposure time after addition of 0.1% formic acid solvent prior to analysis. CID of the isolated m/z 1009 species was performed and data were accumulated from 400 scans to fit the constituent fragments to predict masses. Figure 4A top and bottom panels show the initial spectra (where handling in 0.1% formic acid was minimized) and spectra of the very same sample after 40 min at room temperature. Figure 4B, 4C, and 4D show the spectra of the CID fragments of isolated 1009 m/z species collected after 400 scans, the respective deconvoluted spectral mass assignments, and annotation of the constituent peptide fragments on the His_6_-PA1006 sequence itself. Fig. S2 shows the corresponding fragment masses that enabled construction of the annotations in Fig. 4D. The high resolution fragmentation data showed excellent coverage of the His_6_-PA1006 protein purified from *Pae* and also revealed that it lacks the amino-terminal methionine. In addition, one of the “b” ion fragments is a cleavage between the only two Cys residues (Cys11 and Cys22) of PA1006. When analyzed with Data Analysis, the fragments indicate that Cys11 is not modified, whereas the fragments containing Cys22 are consistent with the addition of a persulfide containing two extra sulfur groups.

### Analysis of His_6_-PA1006 Cys22Ser Mutant Protein by FT-ICR-MS

**Figure 5:**
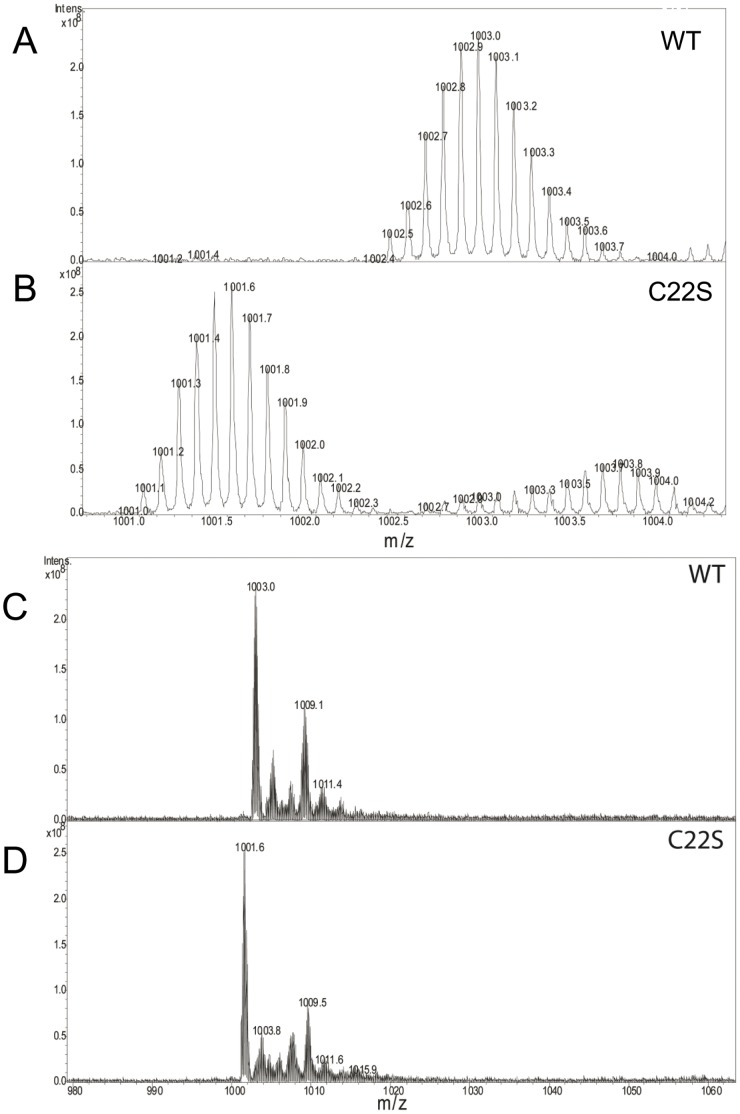
Comparison of FT-ICR-MS spectra of His_6_-PA1006 WT versus Cys22Ser mutant forms. His_6_-PA1006 WT (A and C) and Cys22Ser mutant (B and D) proteins were analyzed by FT-ICR-MS. Panels A and B are zoomed-in views of the spectra in the range of m/z from 1001 to 1004. Shown are the highly resolved isotopic isomers of the WT m/z 1003 (A) and Cys22Ser mutant m/z 1001.6 (B) species that would only be seen as an averaged single peak in lower resolution MS methods. Panels C and D show a broader range of WT (C) and Cys22Ser mutant (D) spectra ranging from m/z 980 to m/z 1060.

To further test the specific modification of Cys22, we expressed and purified a His_6_-PA1006-Cys22Ser mutant protein from *Pae*. Notably, this mutant did not complement the Δ*PA1006* mutant strain even when expressed in high copy number from pEX1.8 after induction with IPTG (data not shown). The mutant protein behaved similarly to WT during purification and the yield and level of purity was also equivalent indicating that activity was not affected by degradation (data not shown). Figure 5 shows a comparison of the FT-ICR-MS spectra of WT His_6_-PA1006 (Panels A and C) versus His_6_-PA1006-Cys22Ser mutant protein (Panels B and D). Notably, the primary (10+) charged species of the His_6_-PA1006-Cys22Ser mutant was m/z 1001.6 (Fig. 5B), which is approximately 16Da decreased in mass compared to the WT m/z 1003 species (Fig. 5A). These data agree nicely with the expected loss of 16 Da that results when oxygen (16 Da) replaces a sulfur (32 Da) atom that occurs when Ser replaces Cys at position 22. Importantly, consistent with the CID fragmentation data presented in Figure 4, the His_6_-PA1006-Cys22Ser mutant does not contain a species that that is +63 Da increased from the 1001.6 m/z parental species, and the spectrum did not change upon reduction with DTT (Fig. 5D and data not shown). These data indicate that, unlike the WT His_6_-PA1006, the His_6_-PA1006-Cys22Ser mutant protein lacks a persulfide modification.

### Analysis of His_6_-PA1006 Treated with Sulfhydryl-reactive N-ethylmaleimide

**Figure 6:**
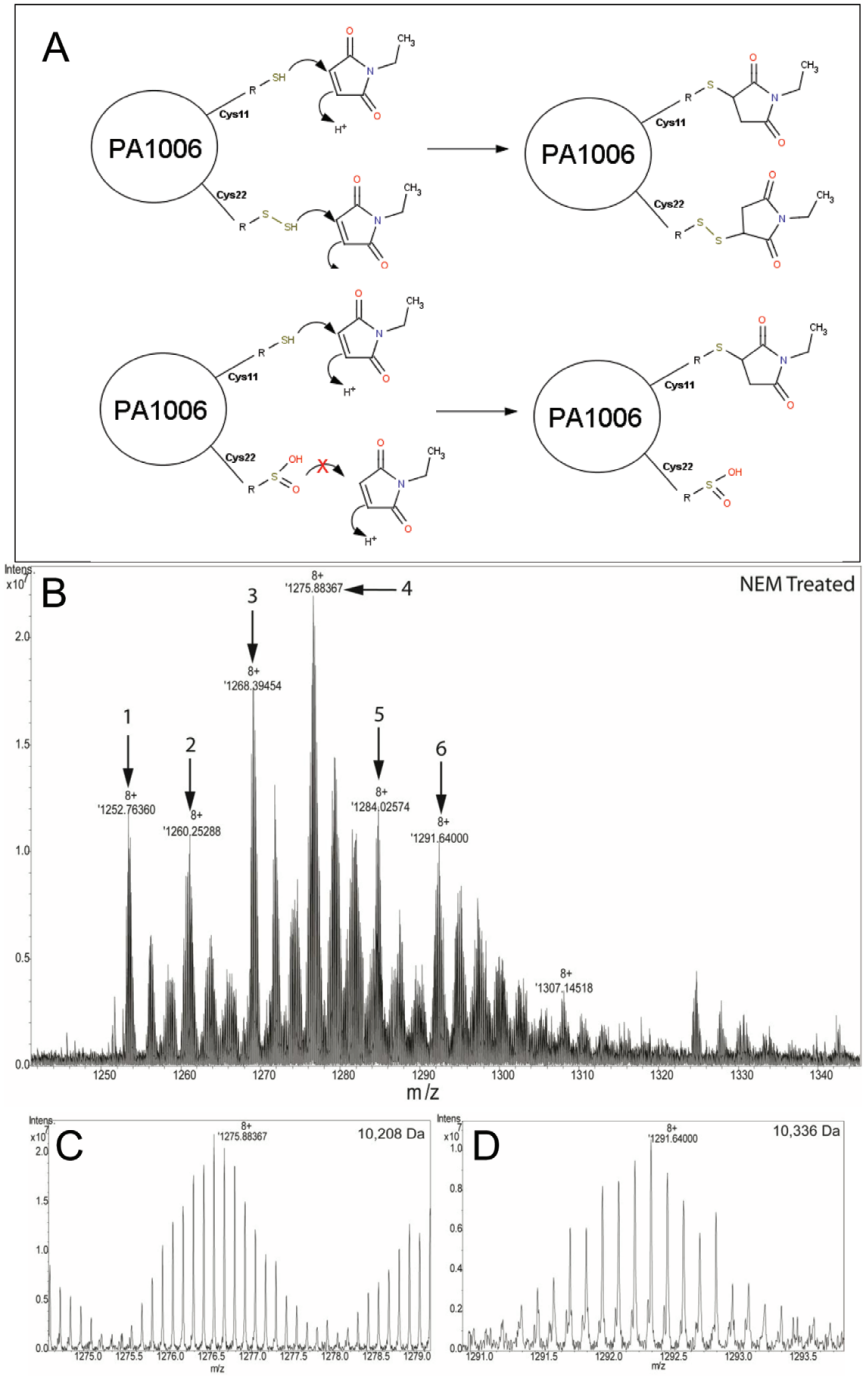
FT-ICR-MS analysis of His_6_-PA1006 treated with N-ethylmaleimide (NEM). A) Predicted reaction of NEM with PA1006 Cys11 and Cys22 (containing the putative persulfide). The top portion of A shows the persulfide form and, in contrast, the bottom portion shows an oxidized form. B) FT-ICR-MS spectrum of His_6_-PA1006 reacted with NEM for 22 h. Species indicated by numbers 1–6 (red) are explained in Figure S2. Panels (C) and (D) show the zoomed-in spectra for species 4 and 6 respectively. The masses of species 4 and 6 are consistent with single or double NEM-adducted forms of His_6_-PA1006 respectively.

Data above suggest that Cys22 is modified in the form of a persulfide; however it is possible that part of the mass change observed by FT-ICR-MS may be due to oxidation of the parental Cys sulfhydryl group or oxidation of persulfide itself [21]. For example, if a single additional sulfur was added to Cys22 (R-S-H) as a persulfide (R-S-SH), it could be oxidized to a sulfinic acid form (R-S-S-O_2_H), and this would be difficult to distinguish from the di-persulfide modification (R-S-S-SH) based upon m/z values alone (see Fig. 6A top and bottom). In addition, a previous report suggests that the *E. coli* PA3667/CsdA may also function as a Cys sulfinate desulfurase [22]. Given the interaction of PA1006 with PA3667/CsdA, the m/z 1009 species of PA1006 purified from *Pae* may reflect either natural oxidation or enzyme catalyzed events. To differentiate between reactive sulfhydryl groups (either Cys sulfhydryl or persulfide forms) versus oxidized persulfide/sulfhydryl groups (which should not react with sulfhydryl-reactive molecule; Fig. 6A top and bottom), we treated His_6_-PA1006 with the sulfhydryl-reactive molecule N-ethylmaleimide (NEM). Figure 6A shows the predicted reactivity of PA1006 Cys11 and Cys22 with NEM if the protein is not oxidized. Since our earlier analyses indicated that the non-conserved Cys11 is not modified, Cys11 was expected to react with NEM. In contrast, reactivity of Cys22 with NEM should depend on the presence of a sulfhydryl or persulfide and be refractory in the case of an oxidized sulfhydryl group. In order to provide the most accurate data, these scans were performed in narrow band mode by FT-ICR-MS with a peak resolution of greater than 100,000. In addition, the observed spectra are the average of 400 scans at this high resolution. We observed several species (Fig. 6B labeled 1–6 in red) after treatment with 4 mM NEM for 22 h. Figure S3 shows the interpretation of each of these species with a cartoon indicating the nature of the species. Figure 6C and 6D show species 4 and 6 more clearly which represent single and double NEM adducts on His_6_-PA1006 consistent with NEM reactivity at both Cys11 and Cys22. Moreover, these data indicate that Cys22 is modified with a persulfide and that the differences in the spectra not due to oxidation.

### PA1006 is Required for Molybdenum Homeostasis

**Figure 7:**
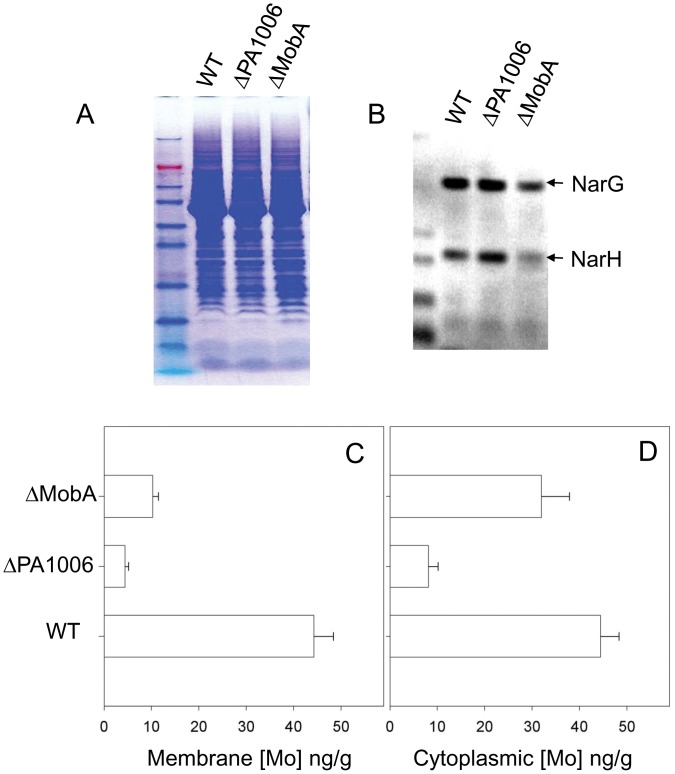
PA1006 affects molybdenum homeostasis. Cytoplasmic and membrane extracts were prepared from *Pae* strains- PAO1 WT, Δ*PA1006*, and Δ*PA3030/mobA*. A) Membrane extracts visualized by SDS PAGE followed by Coomassie staining. B) Western blot of membrane extracts using rabbit α-NarGH antisera as primary antibody. ICP-MS was used to determine molybdenum levels in C) membrane fraction and D) cytoplasmic fraction. Average values of three biological replicates with standard deviation are shown.

Given the loss of nitrate reductase activity in the Δ*PA1006* mutant [9], we hypothesized that the PA1006 persulfide sulfur may contribute to the iron-sulfur clusters (Fe-S) or MoCo sulfur coordination sphere. MoCo is the generic term for a class of sulfurated pterins that bind to (coordinate) a molybdenum atom. Various chemical forms of MoCo are inserted into enzymes (including nitrate reductases) that employ molybdenum at their catalytic sites for electron transfer [23], [24], [25]. Given that the *E. coli* membrane nitrate reductase NarGHI can exist in a non-functional apo form without MoCo, we tested the possibility that MoCo may be absent from the nitrate reductase within the Δ*PA1006* mutant by measuring relative molybdenum levels in the membrane fraction using inductively-coupled-plasma mass spectrometry (ICP-MS). Total membranes prepared from wild-type, Δ*PA1006*, and Δ*PA3030*/*mobA* mutant backgrounds were analyzed for molybdenum content by ICP-MS. The Δ*PA3030*/*mobA* mutant was analyzed in parallel because the MobA protein is required to form the guanine dinucleotide form of MoCo which is utilized by nitrate reductases. Thus, we expected the membrane nitrate reductase NarGHI to be in the apo form (lacking MoCo) in the Δ*PA3030/mobA* mutant and to display a corresponding loss of molybdenum levels in the membrane fraction. Membrane protein yields as well as localization of NarGH nitrate reductase subunits were all similar as assessed by SDS PAGE and western blot (Fig. 7A and 7B). Localization of NarGH in the membrane fraction appears to occur normally in the Δ*PA1006* mutant. ANOVA analysis suggests that each mutant displays significantly different molybdenum concentrations (p<0.006). Notably, the Δ*PA1006* displayed ∼10-fold less molybdenum in its membrane fraction which was even more pronounced than the ∼5-fold reduction displayed by the Δ*PA3030/mobA* mutant (Fig. 7C). Given the dramatic reduction in Mo displayed by the Δ*PA1006* mutant, molybdenum levels of the cytoplasmic fractions of each strain were also determined. The Δ*PA1006* mutant showed a similar (∼10-fold) reduction of molybdenum in the cytoplasmic fraction compared to WT; however, in this case, the Δ*PA3030/mobA* mutant displayed molybdenum levels comparable to WT (Fig. 7D). Cytoplasmic fractions were also analyzed for several other metals (both biological and non-biological). Figure S4 shows the levels of the complete set of metals analyzed for three biological replicates tested. Notably, isotopic lead profiles, which we suggest serve as an internal standard of external contamination, were statistically indistinguishable (P = 0.543) for all cytoplasmic preparations. These data provide confidence that the observed changes in molybdenum levels are significant. Interestingly, other oxyanion forming metals such as vanadate or tungsten (V or W), which are commonly converted into metalloenzyme cofactors similar to MoCo (VCo or WCo) [26] show similar decreases in concentration as molybdenum in the *ΔPA1006* and *ΔPA3030/mobA* mutant strains compared to WT (Figure S4, and data not shown). Only V is reported since W was only determined semi-quantitatively. Nonetheless, side-by-side comparisons show a similar trend to V, with lower [W] as compared to WT.

### PA1006 Interacts with Molybdenum Cofactor Biosynthesis and Nitrate Reductase Proteins

MoCo biosynthesis is a highly orchestrated process involving several enzymes [23], [25]. Given that the intracellular concentration of molybdenum is dramatically reduced in the Δ*PA1006* mutant, we hypothesized that PA1006 protein may interact with MoCo biosynthesis enzymes. To test this hypothesis, we employed a GFP protein fragment complementation assay (GFP-PFCA). GFP-PFCA uses two distinct fragments of the GFP, fused in frame with two potential interacting proteins that will only reassemble to produce a GFP signal if the fused proteins of interest come into close enough proximity in the cell to form a stable fluorescent molecule. This assembled GFP acts as a qualitative interaction “trap” due to the largely irreversible GFP assembly and allows for the detection of weak protein-protein interactions *in vivo*. We adapted a GFP-PFCA system from *E. coli* for *in vivo* investigation in *Pae* [27], [28]. Unlike the yeast-two-hybrid assay, GFP-PFCA offers a more physiologically relevant environment since it is performed in *Pae* itself. For example, GFP-PFCA allows for optimal *Pae* codon usage (since *Pae* genes are generally 60–70% G/C rich). Similarly, if the interaction requires a larger complex for assembly or the action of specific chaperones, performing GFP-PFCA in *Pae* itself will allow this to occur.

**Figure 8:**
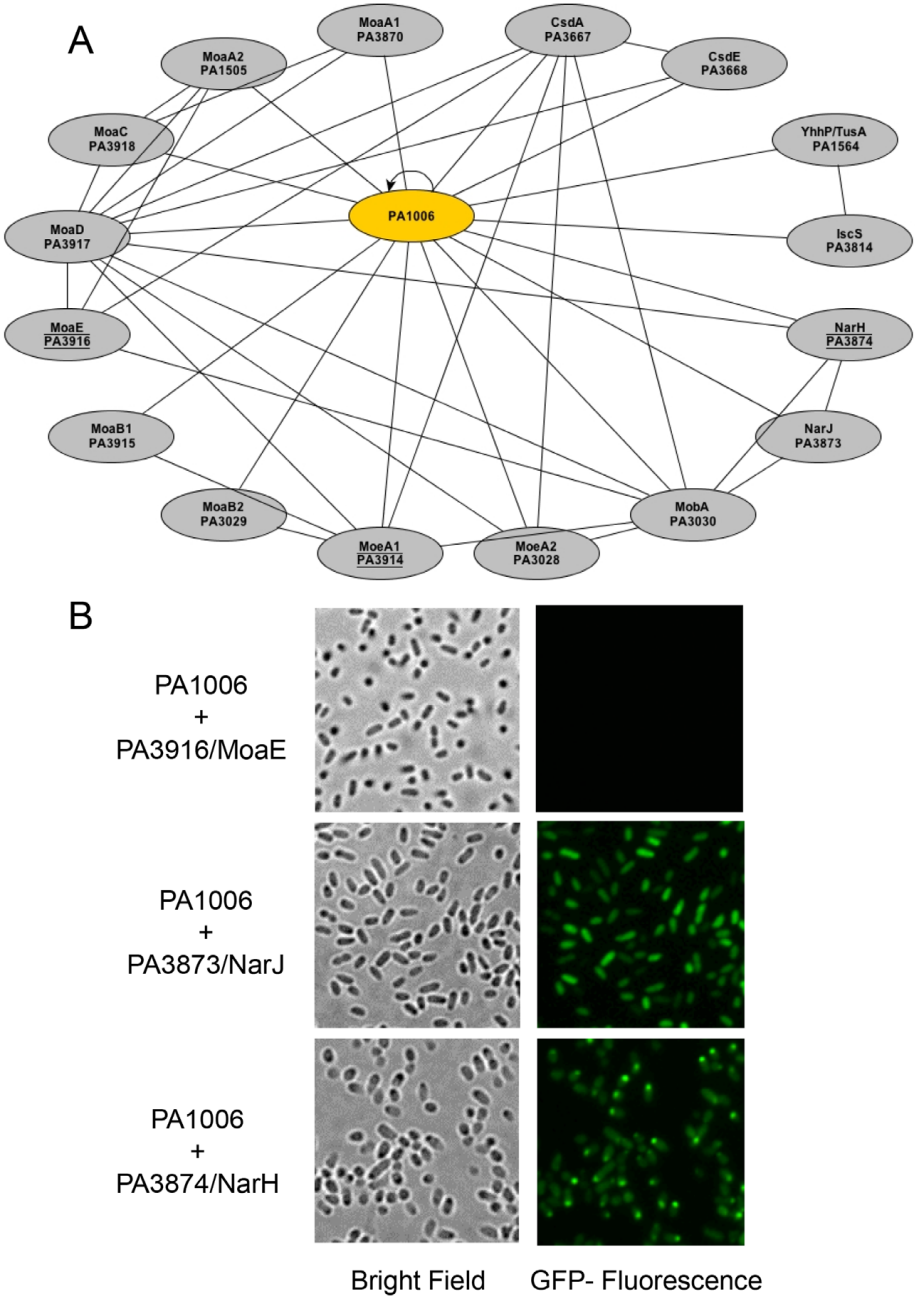
The PA1006 protein interacts with molybdenum cofactor biosynthesis and utilization enzymes. A) Each oval represents a protein evaluated in the GFP-PFCA. A connecting line indicates a positive GFP signal between the pair of proteins, as visualized by fluorescent microscopy. Underlined proteins indicate proteins that appeared localized to one pole of the cell. Network diagram constructed using yEd software. B) Fluorescence microscopy images of GFP-PFCA in *Pae*. Left and right panels show bright field and GFP-fluorescent images (2 second exposures). PA3916/MoaE-PA1006 (no interaction), PA1006-PA3873/NarJ (positive interaction), and PA1006-PA3874/NarH (positive interaction with punctate appearance localized at pole) pairings are shown.

We tested several interactions of PA1006 with MoCo biosynthetic and MoCo requiring enzymes. Figure 8A is a diagram summarizing positive interactions, and Figure 8B shows typical fluorescence observed. MoCo biosynthesis proteins such as PA3917/MoaD, PA3918/MoaC, PA3030/MobA, and both homologs of MoaA (PA3870/MoaA1 and PA1505/MoaA2) and MoeA (PA3914/MoeA1 and PA3028/MoeA2) all showed strong fluorescent signals indicating interactions with PA1006. Interestingly, PA3917/MoaD showed interactions with this entire set as well. As expected from earlier studies of *E. coli* enzymes, including co-crystallization, we also observed a strong interaction between PA3917/MoaD and PA3916/MoaE [29], [30]. While PA3914/MoeA1 interacted with a number of proteins, it did so only when fused with the N-terminal GFP fragment and not the C-terminal fragment. Therefore, a positive GFP signal suggests that the proteins either interact directly in the cell or come into contact with the partner as part of a protein pathway or complex, but a negative signal does not exclude obstruction by the GFP-tag.

Consistent with the yeast-two-hybrid studies, PA1006 also interacted with Cysteine desulfurase components PA3667/CsdA and PA3668/CsdE (Table 1). Interestingly, PA3667/CsdA interacted with many of the other MoCo enzymes. Using GFP-PFCA we were also able to observe an interaction between PA1006 and the cysteine desulfurase PA3814/IscS, which could not be determined with certainty in the yeast system due to a false positive interaction between PA3814/IscS and GAL4-AD itself (Table 1). These data suggest that PA3667/CsdA and PA3814/IscS may each donate a persulfide to PA1006. Redundancy in sulfur donation to PA1006 was further supported by the fact that a PA3667/CsdA transposon mutant (obtained from the University of Washington collection) was able to grow anaerobically with nitrate (data not shown). Anaerobic growth with nitrate was not tested with a PA3814/IscS mutant since deletion of PA3814 appears to confer lethality (zero transposon mutants are available in the entire *Pae* IscS operon).

Most intriguing was that PA1006 appeared to interact with itself, as well as with the closer homolog of *E. coli* YhhP/TusA protein, PA1564/YhhP/TusA. Consistent with previous reports, PA1564/YhhP/TusA showed an interaction with PA3814/IscS [31]. The interaction of PA1006 with PA1564/YhhP/TusA may indicate overlap between sulfur trafficking pathways.

Since PA1006 interacts with MoCo biosynthetic enzymes and the mutant shows reduced molybdenum levels as well as a loss of nitrate reductase activities, we hypothesized that PA1006 may interact with PA3873/NarJ (molybdenum cofactor chaperone) as well as nitrate reductase itself. GFP-PFCA also showed a positive fluorescent signal when PA1006 was paired with either PA3873/NarJ or PA3874/NarH (Fig. 8B). Although *Pae* displays a very low level of intrinsic fluorescence, no GFP fluorescence was detected for negative interactions using the exposure times we employed. For example, a negative interaction between PA3916/MoaE and PA1006 is shown in Figure 8B for reference.

Notably, in all of the PA3874/NarH interactions, the cellular fluorescence signal found by microscopy appeared distinctly punctate at one end of the cell. This intense GFP signal localized to a distinct focal point was also found in PA3916/MoaE and PA3914/MoeA1 interactions. Experiments employing a yellow fluorescent protein- protein fragment complementation assay (YFP-PFCA) in *E. coli* obtained similar microscopic images with proteins known to localize to the membrane [32]. Punctate patterns were only observed when one of the *E. coli* proteins paired was NarH, MoaE, or MoeA1. It is also possible that the punctate patterns we observe indicate that these proteins are part of a larger complex and may indicate that they are membrane tethered. Further studies must be done to address the idea that these proteins are part of a large complex or membrane associated and rule out the possibility that they are nonspecific protein aggregates.

## Discussion

The goal of this study was to determine a function of the PA1006 protein that could account for the biological data (such as loss of nitrate utilization) reported in the companion manuscript [9]. Based upon data in the accompanying manuscript and the homology of PA1006 with the *E. coli* YhhP/TusA protein, we originally hypothesized that: 1) PA1006 Cys22 may be modified as a persulfide *in vivo*, and 2) PA1006 may be connected to molybdenum pathways since nitrate reductases which contain MoCo are inactive and several MoCo biosynthesis genes display altered expression in the Δ*PA1006* mutant [9].

Several pieces of data support the idea that PA1006 is a persulfide-modified protein at Cys22 and that this modification is physiologically relevant. Mutation of the highly conserved Cys22 caused a loss of nitrate reductase activity that appears equivalent to deletion of the entire *PA1006* gene. Yeast-two-hybrid and GFP-PFCA in *Pae* itself each revealed that PA1006 interacts with Cys desulfurase enzymes that mobilize sulfur as a persulfide which can be transferred to downstream protein carriers (such as PA1006 or the *E. coli* YhhP/TusA protein). FT-ICR-MS analyses of His_6_-PA1006 purified from *Pae* itself provided strong direct evidence that Cys22 is modified in the form of a persulfide *in vivo*. It may be interesting to note that we observe two persulfide sulfurs added to PA1006 Cys22. Addition of multiple persulfide sulfur atoms was also reported by Ollagnier-de-Choudens et al.who observed up to four sulfurs added to Cys51 of SufE by SufS [20].

We extended our studies to ascertain how a PA1006 persulfide modification could relate to the loss of nitrate reductase activity as well as connect to molybdenum pathways as suggested by the accompanying study [9]. Given that both nitrate reductase contains Fe-S clusters and MoCo which are coordinated by sulfur that may be derived from persulfides, and the relative rarity of Mo (compared to Fe), we chose to use ICP-MS to investigate Mo levels in the membrane and cytoplasmic fractions. ICP-MS revealed that both membrane and cytoplasmic concentrations of molybdenum are dramatically reduced in the Δ*PA1006* mutant compared to WT. The cytoplasmic data were especially striking considering that all other metals remained nearly equivalent (including Fe). A connection of PA1006 to molybdenum pathways was further bolstered by GFP-PFCA experiments showing interactions of PA1006 with several molybdenum cofactor biogenesis proteins *in vivo*. Given that molybdenum and MoCo are required by nitrate reductase for redox chemistry at its active site, the loss of molybdenum and/or its availability may explain the loss of its activity. Connections between PA1006, molybdenum, and nitrate reductase are also supported by microarray data presented in the companion paper [9]. Genes such as *PA3870/moeA*, and *PA3914-PA3918/moaBCDE*, as well as the membrane nitrate reductase operon *PA3871-PA3877/narGHIJK* were all dramatically up-regulated in the Δ*PA1006* mutant compared to WT when the cells were grown in NY media. Herein we extend these connections by demonstrating that PA1006 protein appears to be in a complex with several MoCo biosynthesis proteins.

A precise mechanistic connection between PA1006-persulfide modification and molybdenum homeostasis is not entirely clear. Since PA1006 interacts with several MoCo biosynthesis enzymes, and is required for nitrate reductase activity in *Pae*, we speculate that PA1006 may function to generally assist with molybdenum utilization or uptake. It is noteworthy that the Δ*PA1006* mutant fails to utilize xanthine or hypoxanthine as a nitrogen source suggesting that xanthine oxidase activity may also be altered [9]. In this case, the Δ*PA1006* mutant would be more severe with respect to Mo-dependent pathways than a *ΔPA3030/*mobA mutant that loses nitrate reductase yet retains xanthine oxidase activity because the guanine dinucleotide (MGD) form of MoCo generated by MobA protein is required by nitrate reductase and not xanthine oxidase. Similarly, the *ΔPA1006* mutant displayed a more general effect on molybdenum homeostasis compared to the *ΔPA3030/mobA* mutant. Therefore, in the *ΔPA1006* mutant, molybdenum or MoCo may not be properly incorporated into various enzymes that require it.

In summary, this study provides evidence that a loss of nitrate reductase activity and other pleiotropic defects displayed by the *ΔPA1006* mutant in the companion paper [9] may stem from a loss of molybdenum-specific enzyme activities which depend on the PA1006 persulfide.

## Methods

### Reagents

Chemicals of the highest grade were purchased from Sigma (St. Louis, MO). All cloning-related oligonucleotide primer sequences are available upon request. N-ethylmaleimide was prepared fresh prior to each experiment.

### Yeast-Two-Hybrid Analysis

Yeast-two-hybrid analysis was performed using the Gal4-based Matchmaker System (Clontech; Mountain View, CA). General methods were followed as indicated in the Yeast Handbook provided by Clontech. The Matchmaker System allows for semi-quantitative assessment of interactions. Growth on synthetic dropout media indicates a relatively strong interaction that supersedes ß-galactosidase activity which is the least stringent measure of interaction. Gal4-DNA binding domain fusion baits were constructed in the vector pGBKT7 and Gal4-transactivation domain prey fusions were constructed in the vector pGADT7. Bait/Prey DNAs were transformed into the *Saccharomyces cerevisiae* strain AH109 using Li Acetate and selected with Leu and Trp. Positive interactions were initially scored after assessing GAL4 activation by measuring growth on synthetic dropout media lacking His (triple dropout; TDO; -Leu/−Trp/−His). If positive for growth on TDO, colonies were also tested for growth on media additionally lacking Ade (quadruple dropout; QDO; -Leu/−Trp/−His/−Ade). We based the score for positive interaction in Table 1 on the ability to at least grow on triple dropout media. All colonies were also tested for weak interactions using the colony lift assay which assays β-galactosidase activity since it is also under control of a GAL4 promoter.

### Cloning, Mutagenesis, Expression, and Purification of PA1006 Protein

#### Mutagenesis and complementation

For complementation studies, wild-type (WT) *PA1006* gene (and its putative promoter) was cloned into pucp18 [33] using PCR followed by subcloning. Subsequently, *PA1006* genes encoding Cys22Ser and Cys22Ala mutant versions were generated in parallel using splice-overlap-extension PCR [34]. In addition, a WT version of *PA1006* encoding for a hexa-histidine tagged form of the protein (His_6_-PA1006) was also generated to test the possibility that the tag may interfere with function. For IPTG-inducible expression in *Pae* the *His_6_*-*PA1006* gene construct was amplified by PCR with engineered flanking amino-terminal/carboxy-terminal *EcoRI/HindIII* (*Pae*) restriction endonuclease cleavage sites, subcloned into pEX1.8 [35] (the complete sequence of pEX1.8 was also determined and entered into Genbank: gi:JQ342676), and the sequence was confirmed.

#### Protein expression and purification

Plasmid pEX1.8-*His_6_-PA1006* was transformed into the Δ*PA1006* mutant PAO1 strain of *Pae* and maintained by selection with 50 µg/ml of carbenicillin. We used the Δ*PA1006* mutant PAO1 strain for all expression studies because we did not want the WT copy of the *PA1006* gene to compete with our construct for persulfide addition (or potential interactions with other proteins that may be required for persulfide addition). Cells (1L) were grown and PA1006 protein was induced with 1 mM IPTG at an optical density of 1.0 at 600 nm. After 4 h induction, cells were harvested by centrifugation and stored in 20 ml of buffer A (50 mM Tris-HCl pH7.4, 300 mM NaCl, 10 mM imidazole, 10% glycerol) at –80°C until purification. For purification, the cell suspension was thawed. Next, protease inhibitor cocktail for His-tagged proteins (Sigma) and a 20 mg/ml stock of lysozyme in buffer A was added to the cells and incubated on ice for 1 h. Cells were lysed by 3 consecutive passages through a French press (SLM-Aminco) at 1000 Psi. Residual cells and membranes were removed by centrifugation at 164,000×g for 1 h at 4°C. An additional equivalent of protease inhibitors were added, and the supernatant containing soluble proteins was rocked at 4°C in a 50 ml conical tube for 4 h with 2 mL of Ni^2+^-NTA agarose beads (Qiagen) that had been washed to remove ethanol and pre-equilibrated with buffer A. The slurry was allowed to pass by gravity flow through a disposable column (Bio-Rad). Beads (with retained proteins) were subsequently washed with an additional 50 mL of buffer A as well as 50 mL of modified buffer A containing 35 mM imidazole. His_6_-PA1006 protein was eluted with 20 ml of modified buffer A containing 500 mM imidazole. Fractions containing His_6_-PA1006 protein as determined by SDS PAGE were pooled and dialyzed against buffer B (50 mM Tris-HCl pH8.0, 10% glycerol, 100 mM NaCl). Next, the sample was resolved by anion exchange MonoQ 5/5 column (GE Life Sciences). The MonoQ column was equilibrated in buffer B, and His_6_-PA1006 protein was recovered in the flowthrough fractions whereas contaminants were retained. Flowthrough fractions were pooled and concentrated using Amicon ultrafiltration spin columns (3K MWCO). His_6_- PA1006 was analyzed by SDS PAGE followed by Coomassie staining and quantitated by its optical density at 280 nm. Analytical gel filtration was performed using an FPLC system with a Superose 6 10/30 column (GE Life Sciences). Protein content of the resolved fractions was quantitated using the Protein Assay Reagent (Bradford Assay)from Bio-Rad which gives an absorbance signal at 595 nm. Data were plotted using a spline with points format using SigmaPlot software.

### Analysis of PA1006 by Mass Spectrometry

#### “Bottom-up” analysis of PA1006

In bottom-up analyses, proteins are first digested or chemically degraded and constituent fragments are examined. Purified PA1006 was digested overnight with trypsin using previously described methods [36]. The sample was then precipitated with two volumes of ethanol, re-suspended in 50 mM ammonium bicarbonate buffer pH 7.5, and analyzed by both matrix-assisted laser desorption ionization-time of flight/time of flight (MALDI-TOF/TOF) mass spectrometry using a AutoFlexIII (Bruker Daltonics) and MicroTofQII, (Bruker Daltonics). MALDI data analyzed using Mascot 2.1.04 (Matrix Science, London, UK) confirmed the peptide as His_6_-PA1006 (data not shown).

#### LC/MS/MS

His_6_- PA1006 was also confirmed bottom-up by LC/MS/MS. LC separation was performed on an Ultimate 3000 (Dionex) plumbed for nanoflow parameters. Separation was done on a 75 µm×15 cm C_18_ PepMap column (3 µm, 100 Å particle size) from LC Packings. Peptides were eluted with a gradient of 5%–50% B over 35 minutes followed by 50%–90% B for 10 minutes at a flow of 400 nL/min MS/MS analysis was performed in-line with the LC on a Micro-TOF QII. MicroTofQII Peptides were identified by searching data against the NCBI nonredundant database for *Pae* proteins with carbamidomethyl cysteine as a fixed modification and oxidized methionine as a variable modification via MASCOT (Matrix Science) [37]. MuDPit scoring was used, and peptides with scores ≥38 were considered to have high homology, or a positive identification (p<0.05). Peptides were accepted if the false-discovery rate was less than 5% and a minimum of one peptide met the identity threshold. All bottom up data were merged and were assembled using ProteinScape (Bruker Daltonics).

#### “Top-down” analysis of PA1006 by FT-ICR-MS

In top-down analyses, intact proteins are examined, and fragmentation can be performed in the MS instrument itself making sequence reconstruction possible. An advantage of this approach is that analysis of the intact protein may allow detection of post-translational modifications which may otherwise be refractory to identification by bottom-up approaches since smaller peptide fragments do not always generate an m/z signal. An aliquot (100 µl) of purified PA1006 (∼40 µM) was passaged twice consecutively through Zeba Spin desalting columns (7K MWCO; Pierce/Thermo Scientific). Next samples were diluted 5-fold with solvent comprised of 60% methanol with either 3% acetic acid or 0.1% formic acid and the final concentration of His_6_-PA1006 protein was routinely in the range of ∼8 µM. Samples were infused at a rate of 180 µL/h and analyzed with a commercial Apex Ultra 9.4T Qh FT-ICR-MS with Apollo II dual source (Bruker Daltonics Inc., Billerica, MA, USA). The FT-ICR instrument was calibrated using standard ESI tuning mixture (part G2421A, Agilent Technologies, Santa Clara CA). Masses were isolated with 10 m/z isolation window, for 100 scans yielding approximately 33000 resolution for m/z 1003 (PA1006 charge state 10+) and m/z 1009 (PA1006 charge state 10+). Fragmentations were accomplished using an m/z 10 isolation window with 17 volts of collision energy for 100 scans. Fragmentation spectra were analyzed using Data Analysis and BioTools (Bruker Daltonics Inc., Billerica, MA, USA). We found that both solvent conditions were effective for a primary His_6_-PA1006 peak (at m/z 1003; +10 charged state); however, a putative persulfide species peak (at m/z 1009; +10 charged state) dissipated rapidly in the 3% acetic acid and required the 0.1% formic acid solvent to be seen during the timescale of the experiments (Fig. 2D and data not shown).

### Molybdenum and Other Metal Analysis by ICP-MS

#### Preparation of extracts

Starter cultures were grown overnight in LB and diluted the following morning into NY media supplemented with 100 mM KNO_3_ to a final optical density at 600 nm (OD_600_) of ∼0.05 (500 ml in 2L baffled flasks). Cells were grown shaking at 37°C and harvested when their optical density at 600 nm (O.D.600 nm) ∼1.0. Cells were pelleted by centrifugation 6300×g at 4°C for 15 min, washed once with 100 ml of 1× PBS, and centrifuged as previously. Next, the wet weights of the cell pellets were determined and the cells were finally resuspended in 1×PBS at 1 g/2 ml, frozen rapidly on dry ice, and stored at –80°C. Crude membrane and cytoplasmic extracts were prepared as follows. Cells were thawed, and protease inhibitor cocktail for bacterial cell extracts (Sigma) was added at the prescribed concentration and lysozyme was added at a final concentration of 1 mg/ml. Cells were stored on ice for 1.5 h and gently inverted every 15 min. Next, cells were disrupted by sonication (Branson sonicator with a microtip) on ice by 3×20 s pulses with ∼3 min between each pulse to assure that heating did not occur. Disrupted cells were centrifuged at 6300×g at 4°C for 15 min to remove residual intact cells. Lysates were then centrifuged at 192,342×g for 70 min at 4°C. The supernatant from this spin was quickly transferred to a clean tube on ice and then aliquotted into tubes placed on dry ice. Pellets, or the membrane fraction, were resuspended in 2 ml ice cold 18MΩ-pure distilled and deionized water (ddH_2_O) by vortexing/pipetting taking care to mix well-and avoiding bubbles and then centrifuged again at 192,342×g for 70 min at 4°C. Membrane pellets were resuspended in 150 µl ice cold 18 MΩ-pure ddH_2_O, and frozen on dry ice. Membrane and cytosolic extracts were stored at –80°C until ICP-MS analysis. Extracts were also analyzed by SDS PAGE and western blot using rabbit polyclonal α-NarGH antisera as the primary antibody (gift of Axel Magalon; CNRS, France). Total protein content was quantitated by the BCA method (Pierce/Thermo).

#### Handling of extracts for ICP-MS analysis

Sample preparation for ICP-MS was conducted in a Class 100 trace metal free clean laboratory in the Environmental Earth and Ocean Sciences Department at the University of Massachusetts-Boston. Prior to opening and preparation, samples were allowed to equilibrate with room temperature for approximately 4 h. After samples had equilibrated with room temperature, samples were agitated with a GlobalSpec Laboratory shaker (Troy, NY USA) for 30 min followed by 1 min of vortex shaking to re-homogenize the fluid. Each sample vial was prepared and diluted in a trace metal clean, class A polypropylene test tube using manufacturer calibrated and validated Eppendorf pipettes. Sample dilutions were prepared volumetrically (and validated gravimetrically) by adding trace metal free concentrated (15.9 mol/L) ultra-pure nitric acid (HNO_3_) (Fisher Scientific, Mass, USA) and then diluted using 18.2 MΩ cm ddH_2_O. Internal standards consisting of known quantities of indium (In) and bismuth (Bi), used to correct for instrumental drift, were added to the samples. Sample dilution resulted in a final concentration of 2% nitric acid (by volume), and 5 ng/g of the internal standards In and Bi, respectively. After re-homogenization was complete, sample vials were labeled, and sample dilutions were prepared, 0.1 mL of each sample was volumetrically pipetted into the corresponding pre-labeled analytical vials to result in a total dilution factor of 10∶1. At time of sample pipette transport, sample mass was verified gravimetrically as weights were recorded to ±0.001 mg. All standards used during ICP-MS analysis, including standards, procedural blanks, and interference check standards were prepared in an analogous fashion.

#### Analytical procedures and uncertainty

Molybdenum (Mo) concentrations were measured using a Perkin Elmer Axiel Field Technology DRC II inductively coupled plasma mass spectrometer (ICP-MS). Prior to sample analysis, instrumentation parameters were optimized for sensitivity and stability by using a multi-element tuning solution containing 5 ng/g Be, Mg, Zn, Mo, In, Ba, Ce, Bi, and U to minimize the formation of doubly charged species (e.g. Ba^2+^/Ba^+^<1.5%) and oxides (CeO^+^/Ce^+^ <1.5%). Solution based ICP-MS analyses were conducted with modifications to the EPA 6020A methodologies [38], [39] the approved ICP-MS analytical procedure for inorganic trace elements in soils and sediments. This analytical method is analogous to the methods for the analysis of metals in biological fluids, tissue, and bone [40] Optimization was conducted prior to each batch of analysis. To clean sample lines and reduce memory effects, sample lines were washed sequentially with 18.2 MΩ cm ddH_2_O for 75 s and a 2% nitric acid solution for an additional 75 s between analyses. Procedural blanks and interference check standards were analyzed before and after each analysis, to monitor and correct for instrumental and procedural backgrounds and isobaric interferences respectively. Seven calibration standards used to determine Mo concentrations in serum samples included aliquots of 18.2 MΩ ddH_2_O spiked with known quantities of Mo in a linear range from 0.100 ng/g to 25 ng/g. Calibration standards were prepared from 1,000 mg/L Mo single element standards (SCP Science, USA). Aliquots with known concentrations of Mo solutions (prepared separately from calibrations standards) were analyzed as unknowns to determine external precision as less than 3%. Isobaric corrections were performed on-line using ICP-MS software. Ten duplicate analyses (n = 10) were performed for all analytes for each sample solution. Limits of detection (LOD) according to Long and Winefordner [41] are as follows: Mo = 0.011 pg/g (ppt). Limits of quantification (LOQ) according to Long and Winefordner [41] are as follows: Pt = 0.035 pg/g (ppt). Method detection limits (MDL) were calculated according to the two-step approach using the t99SLLMV method [42] at 99% CI (t = 3.71) as Pt = 0.011 pg/g (ppt). Because MDL values incorporate matrix effects and interferences associated with the complex, biomolecule-rich sample matrix, we suggest MDLs provide a more robust and accurate limit of detection for these analyses.

### Protein Fragment Complementation Assay (PFCA) in Pseudomonas Aeruginosa

The PFCA approach using two halves of green fluorescent protein (GFP) pioneered by the Regan Lab [27], [28] for detecting protein-protein interactions in *E. coli* was adapted to detect protein-protein interactions *in vivo* in *Pae*. Previously constructed N-terminal (NGFP) and C-terminal (CGFP) GFP–link vectors [27], [28], containing a small multiple cloning site (MCS) for gene insertion linked to an amino acid spacer and cleaved GFP sequence, were used in this assay. The NGFP construct was placed under *Ptac* promoter in pEX1.8, an *E. coli*/*Pae* shuttle vector, using primers *EcoRI* 5′-GAATTCGAGCGGATAACAATTCCCC-3′ and a *PstI* 5′-CTGCAGCAGCAGCCAACTCAGCTTC-3′. The CGFP-link construct, including the araC transcriptional activator of the parent *E. coli* plasmid, pMRBAD-link-CGFP, to allow expression under the arabinose promoter (PBAD), was inserted into mini-Tn7T-GM using *SacI* 5′-GAGCTCAATTATGACAACTTGACGGCTAC-3′ and *KpnI* 5′-GGTACCTTTCAGCAAAAAACCCCTC-3′ [27], [28]. Genes of interest were cloned in frame into the MCS downstream of NGFP, into the *XhoI* and *XmaI* sites, and upstream of CGFP, into the *NcoI* and *SphI* sites eliminating the gene of interest’s stop codon, using gene specific primers amplifying the coding sequence. The resulting plasmids were all sequenced confirmed before introduction into *Pae*. The CGFP-tagged gene was inserted into a unique location downstream of the *Pae glmS* gene by electroporation using pTNS2 to facilitate chromosomal insertion [43], [44]. The NGFP-tagged gene was introduced *in trans* via electroporation. Resulting strain were verified by PCR to access the presence of GFP-tagged genes. Strains were grown either on solid or in liquid LB media, containing 0.1% arabinose, 100 µM IPTG and antibiotic pressure, overnight at 37°C followed by 2–4 days at room temperature. Positive GFP interactions were scored based on visual inspection for GFP using an Olympus U-MNB filter (excitation at 470–490 nm, emission at 520 nm) and an Olympus BX41 microscope.

## Supporting Information

Figure S1 **Purification of recombinant PA1006 and PA3667 from** ***E. coli*** **and** ***in vitro*** **sulfur transfer assay.** SDS-PAGE analysis of (A) His_6_-PA1006 and (B) His_6_-PA3667/CsdA from *E. coli.* Proteins were cloned into pET24D (Novagen), expressed in BL21pLysS, and purified to near homogeneity by Ni^2+^-NTA agarose (Qiagen) followed by MonoQ (GE Life Sciences). Two micrograms were loaded into each lane. (C) Absorption spectrum of purified His_6_-PA3667 showing a ∼400 nm species that can be reduced with sodium borohydride treatment indicating the presence of a pyridoxal phosphate cofactor (inset). (D) Analytical gel filtration analysis (Superose 6;-GE Life Sciences) of His_6_-PA3667 shows a monodisperse species that approximates the size of a dimer (∼100 kDa). (E) In vitro sulfur transfer assay. Reactions (30 µl) containing 50 µM ^35^S Cys (100Ci/mmol) in 50 mM Tris-HCl pH 7.5, 100 mM NaCl, 2 µM His_6_-PA3667, and if present, 10 µM His_6_-PA1006 were incubated for 10 min at 37°C and then resolved by a 4–20% gradient SDS-PAGE under non-reducing conditions (and samples were not boiled prior to loading). Gels were visualized with a phosphorimager (Molecular Devices) using Image Quant software. Lane 1 shows PA3667/CsdA+PA1006, lane 2 shows PA1006 alone, and lane 3 shows PA3667/CsdA alone. Note: A similar signal was observed if PA1006 was reduced with DTT (and subsequently removed by passage through a Zeba Column- Thermo/Pierce) prior to the reaction. This suggests that a pre-existing persulfide did not prevent sulfur transfer to PA1006.
(TIF)

Figure S2 **Constituent peptide fragments of His_6_-PA1006** **m/z 1009 species isolated and fragmented by CID in the FT-ICR-MS.** This shows the peak m/z assignments for the m/z 1009 species of the His_6_-PA1006 protein that was isolated and fragmented by CID in the FT-ICR-MS. These data correspond to the annotated diagram shown in Fig. 4D.
(TIF)

Figure S3 **Interpretation of FT-ICR-MS data of His_6_-PA1006 treated with N-ethylmaleimide (NEM).** Molecular forms associated with a particular m/z species were interpreted from data in Figure 6 using a mass of 125 Da for NEM.
(TIF)

Figure S4 **Complete metal analysis report as determined by ICP-MS for PAO1 WT,** ***ΔPA3030/mobA*** **, and** ***ΔPA1006*** **mutant strains.** Shown are the results obtained for each metal for three biological replicates (independently grown and processed cultures) of each strain. Metal values were from the cytoplasmic fraction prepared from each strain.
(TIF)
