# Supplementary figures and images for: Pseudomonas aeruginosa PA1006 Is a Persulfide-Modified Protein That Is Critical for Molybdenum Homeostasis
Source: PLoS One. 2013 Feb 8;8(2):e55593. doi: 10.1371/journal.pone.0055593 (PMC3568144; doi:10.1371/journal.pone.0055593)

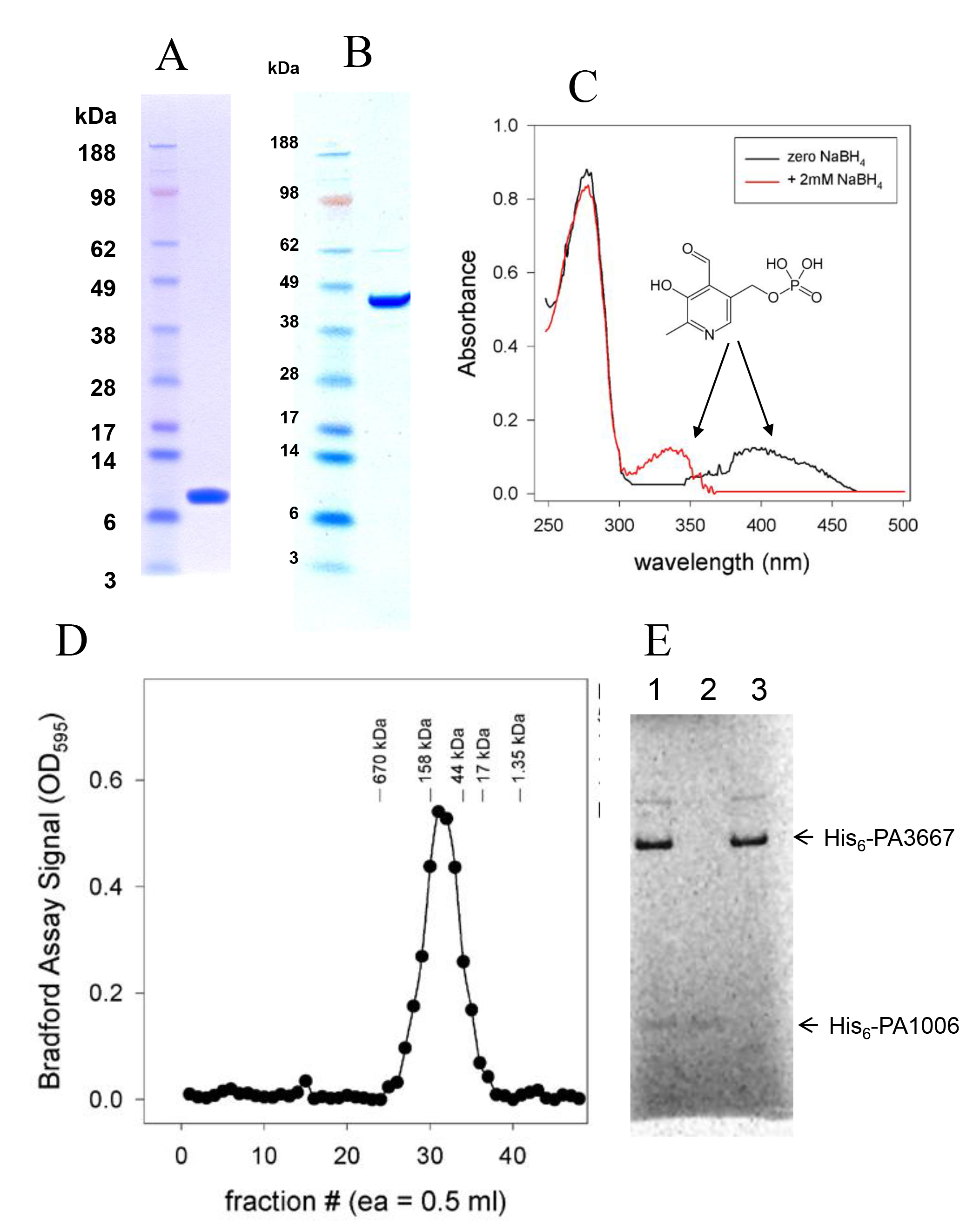

Supplement: Figure S1 — Purification of recombinant PA1006 and PA3667 from E. coli and in vitro sulfur transfer assay. SDS-PAGE analysis of (A) His6-PA1006 and (B) His6-PA3667/CsdA from E. coli. Proteins were cloned into pET24D (Novagen), expressed in BL21pLysS, and purified to near homogeneity by Ni2+-NTA agarose (Qiagen) followed by MonoQ (GE Life Sciences). Two micrograms were loaded into each lane. (C) Absorption spectrum of purified His6-PA3667 showing a ∼400 nm species that can be reduced with sodium borohydride treatment indicating the presence of a pyridoxal phosphate cofactor (inset). (D) Analytical gel filtration analysis (Superose 6;-GE Life Sciences) of His6-PA3667 shows a monodisperse species that approximates the size of a dimer (∼100 kDa). (E) In vitro sulfur transfer assay. Reactions (30 µl) containing 50 µM 35S Cys (100Ci/mmol) in 50 mM Tris-HCl pH 7.5, 100 mM NaCl, 2 µM His6-PA3667, and if present, 10 µM His6-PA1006 were incubated for 10 min at 37°C and then resolved by a 4–20% gradient SDS-PAGE under non-reducing conditions (and samples were not boiled prior to loading). Gels were visualized with a phosphorimager (Molecular Devices) using Image Quant software. Lane 1 shows PA3667/CsdA+PA1006, lane 2 shows PA1006 alone, and lane 3 shows PA3667/CsdA alone. Note: A similar signal was observed if PA1006 was reduced with DTT (and subsequently removed by passage through a Zeba Column- Thermo/Pierce) prior to the reaction. This suggests that a pre-existing persulfide did not prevent sulfur transfer to PA1006. (TIF) [file pone.0055593.s001.tif]

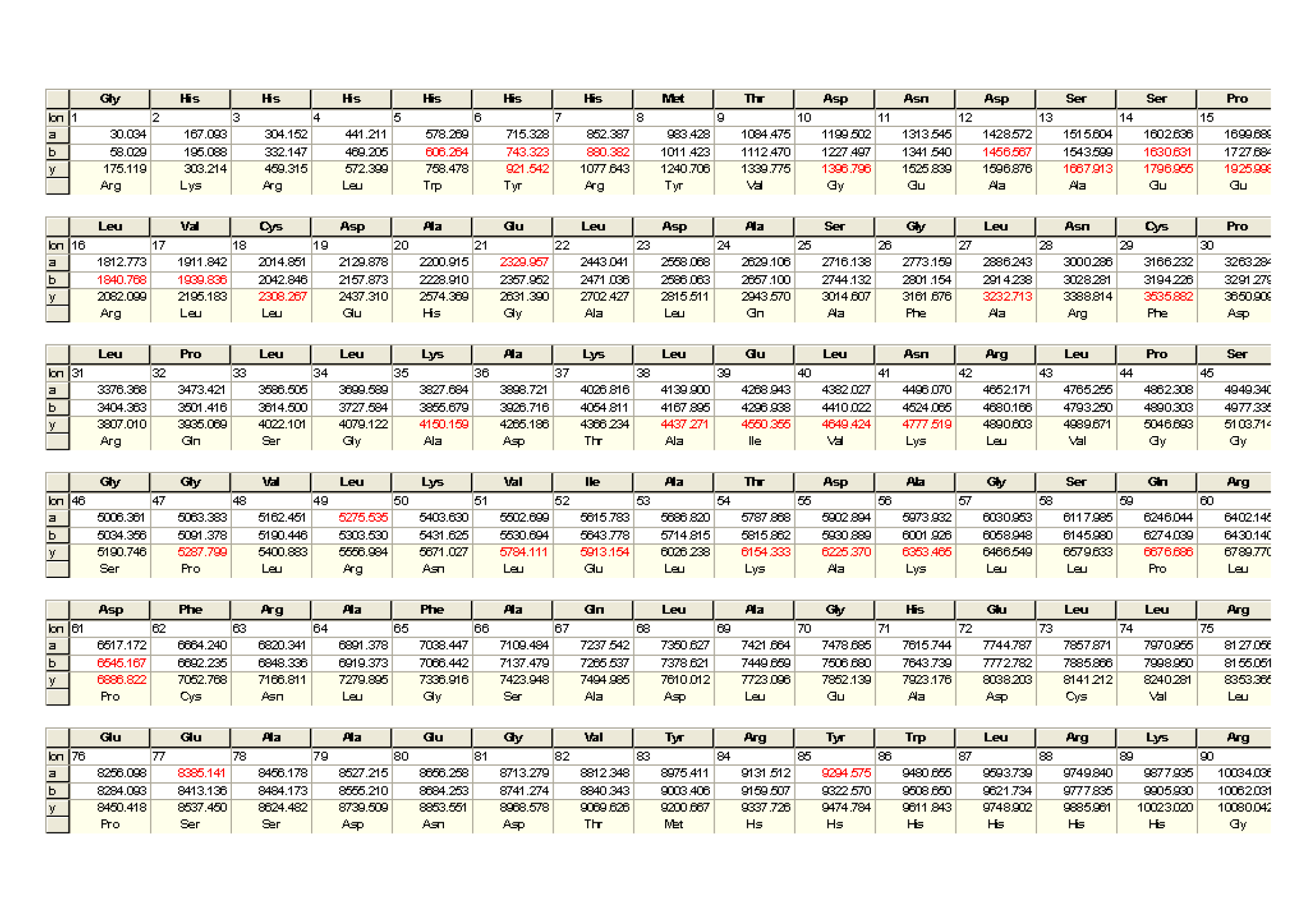

Supplement: Figure S2 — Constituent peptide fragments of His6-PA1006 m/z 1009 species isolated and fragmented by CID in the FT-ICR-MS. This shows the peak m/z assignments for the m/z 1009 species of the His6-PA1006 protein that was isolated and fragmented by CID in the FT-ICR-MS. These data correspond to the annotated diagram shown in Fig. 4D. (TIF) [file pone.0055593.s002.tif]

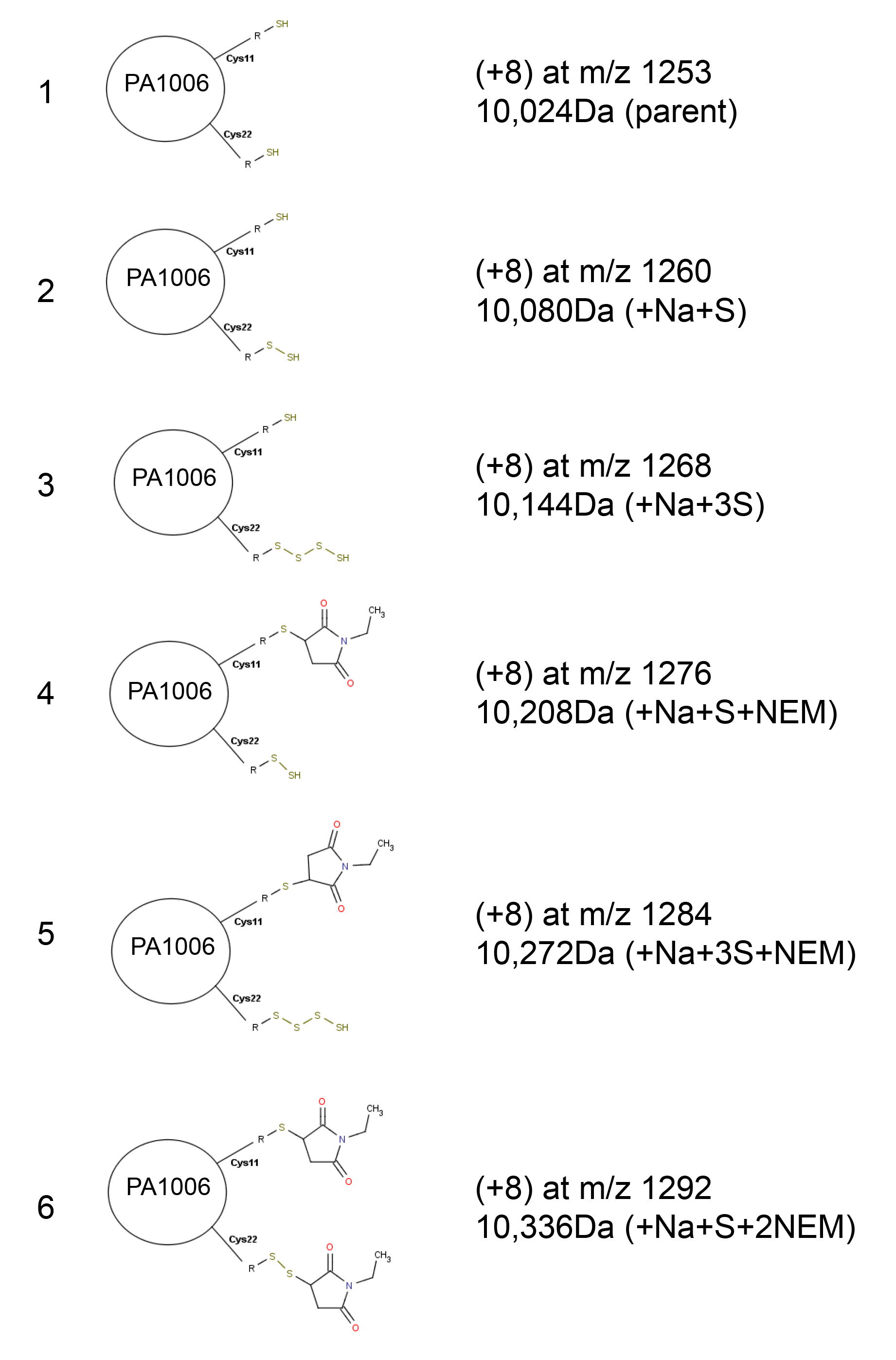

Supplement: Figure S3 — Interpretation of FT-ICR-MS data of His6-PA1006 treated with N-ethylmaleimide (NEM). Molecular forms associated with a particular m/z species were interpreted from data in Figure 6 using a mass of 125 Da for NEM. (TIF) [file pone.0055593.s003.tif]

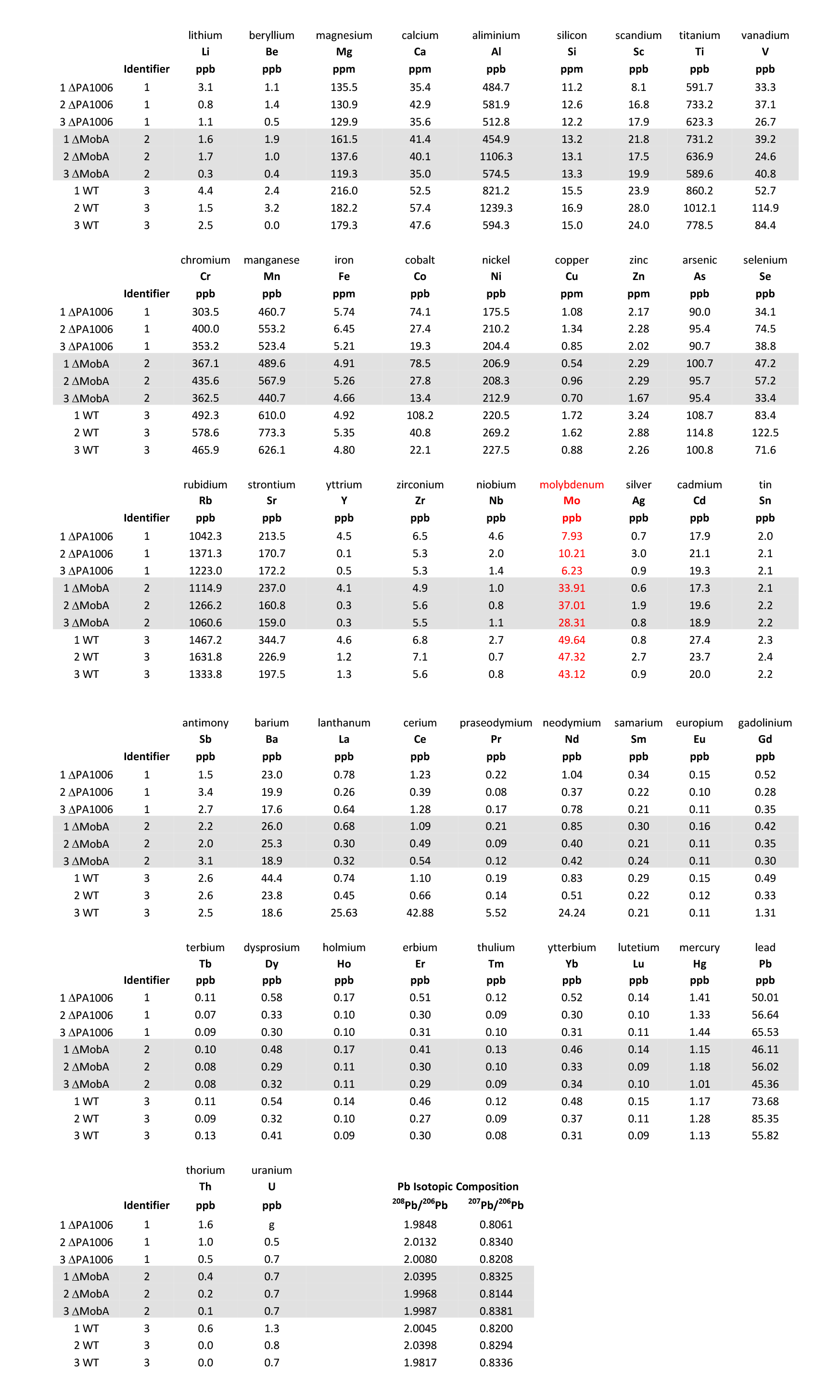

Supplement: Figure S4 — Complete metal analysis report as determined by ICP-MS for PAO1 WT, ΔPA3030/mobA , and ΔPA1006 mutant strains. Shown are the results obtained for each metal for three biological replicates (independently grown and processed cultures) of each strain. Metal values were from the cytoplasmic fraction prepared from each strain. (TIF) [file pone.0055593.s004.tif]
